# Bis(μ-2-carb­oxy-5-nitro­benzoato-κ^2^ *O* ^1^:*O* ^1^)bis­[(2,2′-bipyridine-κ^2^ *N*:*N*′)chloridocopper(II)] dihydrate

**DOI:** 10.1107/S1600536811046782

**Published:** 2012-01-07

**Authors:** Hui Wang

**Affiliations:** a Department of Chemistry, Mudanjiang Normal College, Mudanjiang 157012, People’s Republic of China

## Abstract

The asymmetric unit of the title complex, [Cu_2_(C_8_H_4_NO_6_)_2_Cl_2_(C_10_H_8_N_2_)_2_]·2H_2_O, contains two half binuclear complex molecules and two solvent water molecules; the complete complex molecule is generated by the application of a centre of inversion in each case. Each independent Cu^II^ cation is penta-coordinated within a distorted square-pyramidal environment defined by a two μ_2_-O atoms (derived from two 2-carboxy-5-nitrobenzoato anions), two N atoms (bipyridine ligand) and one Cl. Binuclear species are assembled into a two-dimensional supramolecular architecture parallel to (01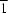) by O—H⋯O and O—H⋯Cl hydrogen bonds.

## Related literature

For an introduction to coordination polymers, see Chen *et al.* (2001 ▶); Wang *et al.*(2009*b* ▶). For a related structure, see: Wang (2009*a* ▶). 
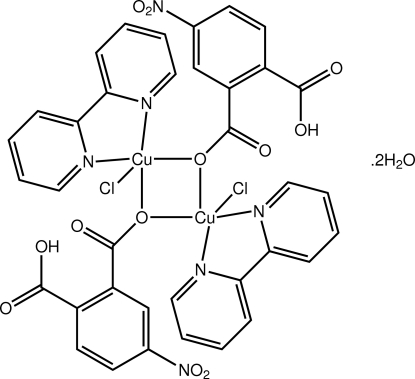


## Experimental

### 

#### Crystal data

[Cu_2_(C_8_H_4_NO_6_)_2_Cl_2_(C_10_H_8_N_2_)_2_]·2H_2_O
*M* *_r_* = 966.62
Triclinic, 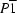

*a* = 9.1090 (12) Å
*b* = 12.3571 (17) Å
*c* = 17.024 (2) Å
α = 92.684 (2)°
β = 101.551 (2)°
γ = 92.493 (2)°
*V* = 1872.6 (4) Å^3^
*Z* = 2
Mo *K*α radiation
μ = 1.36 mm^−1^
*T* = 293 K
0.12 × 0.10 × 0.08 mm

#### Data collection

Bruker SMART CCD area-detector diffractometer
Absorption correction: multi-scan (*SADABS*; Sheldrick, 2003 ▶) *T*_min_ = 0.854, *T*_max_ = 0.899
13021 measured reflections
6505 independent reflections
4541 reflections with *I* > 2σ(*I*)
*R*_int_ = 0.036

#### Refinement

*R*[*F* ^2^ > 2σ(*F* ^2^)] = 0.052
*wR*(*F* ^2^) = 0.149
*S* = 1.05
6505 reflections
555 parameters
12 restraints
H atoms treated by a mixture of independent and constrained refinement
Δρ_max_ = 1.27 e Å^−3^
Δρ_min_ = −0.47 e Å^−3^

### 

Data collection: *SMART* (Bruker, 2005 ▶); cell refinement: *SAINT-Plus* (Bruker, 2005 ▶); data reduction: *SAINT-Plus*; program(s) used to solve structure: *SHELXS97* (Sheldrick, 2008 ▶); program(s) used to refine structure: *SHELXL97* (Sheldrick, 2008 ▶); molecular graphics: *XP* in *SHELXTL* (Sheldrick, 2008 ▶) and *DIAMOND* (Brandenburg, 2005 ▶); software used to prepare material for publication: *SHELXL97*.

## Supplementary Material

Crystal structure: contains datablock(s) I, global. DOI: 10.1107/S1600536811046782/im2329sup1.cif

Structure factors: contains datablock(s) I. DOI: 10.1107/S1600536811046782/im2329Isup2.hkl

Additional supplementary materials:  crystallographic information; 3D view; checkCIF report

## Figures and Tables

**Table 1:** Hydrogen-bond geometry (Å, °)

*D*—H⋯*A*	*D*—H	H⋯*A*	*D*⋯*A*	*D*—H⋯*A*
O3—H3*A*⋯O14^i^	0.82	1.87	2.664 (6)	164
O12—H12*A*⋯O13	0.82	2.03	2.611 (8)	127
O13—H1*W*⋯O14	0.83 (1)	2.64 (5)	3.229 (7)	129 (5)
O13—H2*W*⋯Cl1^ii^	0.84 (1)	2.34 (2)	3.168 (5)	169 (8)
O14—H3*W*⋯O11	0.83 (1)	2.20 (3)	2.992 (7)	158 (8)
O14—H4*W*⋯O5^ii^	0.83 (1)	2.30 (5)	3.010 (6)	143 (7)

Symmetry codes: (i) 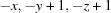; (ii) 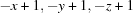.

**Fig. 1.**
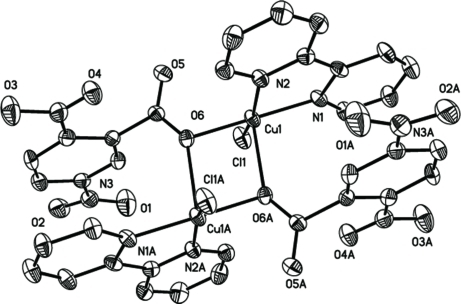
Molecular structure of the title compound. Displacement ellipsoids are drawn at the 30% probability level.

**Fig. 2.**
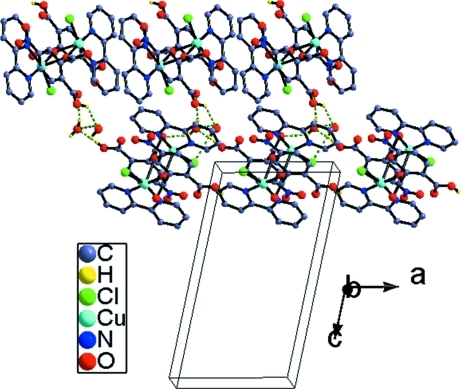
A view of the supramolecular structure of (I) (Brandenburg, 2005).

## supplementary crystallographic information

### Comment

In the field of supramolecular chemistry and crystal engineering, the design and synthesis of coordination polymers have been emerging as an ongoing field owing to their structural aesthetics and topologies as well as diverse functional properties (Chen *et al.*, 2001). Thus far, significant advance achieved in this field has led to a lot of promising materials through the self-assembly of organic ligands and metal ions. Nevertheless, it still remains a great and long-term challenge to exactly predict the molecular structure and functional properties of coordination polymers because of many subtle factors involved in the crystallization process (Wang *et al.*, 2009*b*). As an extension of our work focusing on the assembly of the mixed ligands in the presence of metal ions, the title compound (I) was synthesized and characterized by *x*-ray diffraction (Fig. 1).

Compound (I) crystallizes in the triclinic system with two half complex binuclear molecules and two water molecules in the asymmetric unit. Each copper(II) ion is penta-coordinated exhibiting a distorted square-pyramidal coordination sphere. Cu—O and Cu—N bond lengths are in the normal range if compared with those of reported compounds containing O—Cu—N segments (Wang, 2009*a*). Adjacent dinuclear species are assembled into a two-dimensional supramolecular framework by O–H···O and O–H···Cl hydrogen bonds (Fig. 2).

### Experimental

A mixture of CuCl_2_ (0.027 g, 0.2 mmol), 2,2'-bipyridine (0.032 g, 0.2 mmol), 4-nitro-phthalic acid (0.042 g, 0.2 mmol), and H_2_O (15 ml) was sealed in a 25 ml Teflon-lined stainless steel reactor which was heated to 115°C. Blue block-shaped crystals suitable for X-ray diffraction analysis were separated by filtration (yield: 0.023 g, 24% based on 4-nitro-phthalic acid).

### Refinement

All non-solvate and non-carboxy H atoms were placed in geometrically idealized positions and treated as riding on their parent atoms with C—H = 0.93 Å, *U*_iso_ = 1.2U_eq_ (C) for aromatic atoms. The command '*DFIX*' has been used to restrain the distance of H—O in the water solvate and carboxyl groups as well as bonds C19—C24 and C24—C23. The '*DELU*' instruction has been used to restrain the displacement parameters of C19, C24, and C23) .

### Crystal data

**Table:**

[Cu_2_(C_8_H_4_NO_6_)_2_Cl_2_(C_10_H_8_N_2_)_2_]·2H_2_O	*Z* = 2
*M**_r_* = 966.62	*F*(000) = 980
Triclinic, *P*1	*D*_x_ = 1.714 Mg m^−^^3^
Hall symbol: -P 1	Mo *K*α radiation, λ = 0.71073 Å
*a* = 9.1090 (12) Å	Cell parameters from 2695 reflections
*b* = 12.3571 (17) Å	θ = 2.3–22.0°
*c* = 17.024 (2) Å	µ = 1.36 mm^−^^1^
α = 92.684 (2)°	*T* = 293 K
β = 101.551 (2)°	Block, blue
γ = 92.493 (2)°	0.12 × 0.10 × 0.08 mm
*V* = 1872.6 (4) Å^3^	

### Data collection

**Table:**

Bruker SMART CCD area-detector diffractometer	6505 independent reflections
Radiation source: fine-focus sealed tube	4541 reflections with *I* > 2σ(*I*)
graphite	*R*_int_ = 0.036
Detector resolution: 0 pixels mm^-1^	θ_max_ = 25.0°, θ_min_ = 2.1°
phi and ω scans	*h* = −10→10
Absorption correction: multi-scan (*SADABS*; Sheldrick, 2003)	*k* = −14→14
*T*_min_ = 0.854, *T*_max_ = 0.899	*l* = −20→20
13021 measured reflections	

### Refinement

**Table:**

Refinement on *F*^2^	Primary atom site location: structure-invariant direct methods
Least-squares matrix: full	Secondary atom site location: difference Fourier map
*R*[*F*^2^ > 2σ(*F*^2^)] = 0.052	Hydrogen site location: inferred from neighbouring sites
*wR*(*F*^2^) = 0.149	H atoms treated by a mixture of independent and constrained refinement
*S* = 1.05	*w* = 1/[σ^2^(*F*_o_^2^) + (0.0779*P*)^2^ + 0.7569*P*] where *P* = (*F*_o_^2^ + 2*F*_c_^2^)/3
6505 reflections	(Δ/σ)_max_ = 0.041
555 parameters	Δρ_max_ = 1.26 e Å^−^^3^
12 restraints	Δρ_min_ = −0.47 e Å^−^^3^

### Special details

**Table:**

Geometry. All e.s.d.'s (except the e.s.d. in the dihedral angle between two l.s. planes) are estimated using the full covariance matrix. The cell e.s.d.'s are taken into account individually in the estimation of e.s.d.'s in distances, angles and torsion angles; correlations between e.s.d.'s in cell parameters are only used when they are defined by crystal symmetry. An approximate (isotropic) treatment of cell e.s.d.'s is used for estimating e.s.d.'s involving l.s. planes.
Refinement. Refinement of *F*^2^ against ALL reflections. The weighted *R*-factor *wR* and goodness of fit *S* are based on *F*^2^, conventional *R*-factors *R* are based on *F*, with *F* set to zero for negative *F*^2^. The threshold expression of *F*^2^ > σ(*F*^2^) is used only for calculating *R*-factors(gt) *etc*. and is not relevant to the choice of reflections for refinement. *R*-factors based on *F*^2^ are statistically about twice as large as those based on *F*, and *R*- factors based on ALL data will be even larger.two quite high residual electron densityExplainThe two high residual Q peaks with electron density with 1.27 and 1.22, respectively, are located near the 4-nitrophthalic acid framework, which may be the ghost peaks. This is possible caused due to the poor crystal quality.

### Fractional atomic coordinates and isotropic or equivalent isotropic displacement parameters (Å^2^)

**Table:**

	*x*	*y*	*z*	*U*_iso_*/*U*_eq_	
C1	0.5338 (6)	0.0982 (4)	1.0871 (3)	0.0387 (12)	
C2	0.4007 (7)	0.0423 (5)	1.0863 (3)	0.0497 (15)	
H2	0.3943	−0.0140	1.1200	0.060*	
C3	0.2752 (7)	0.0735 (4)	1.0329 (3)	0.0479 (14)	
H3	0.1833	0.0365	1.0310	0.057*	
C4	0.2821 (6)	0.1574 (4)	0.9825 (3)	0.0403 (12)	
C5	0.4219 (6)	0.2133 (4)	0.9861 (3)	0.0346 (11)	
C6	0.5477 (6)	0.1831 (4)	1.0392 (3)	0.0398 (12)	
H6	0.6402	0.2198	1.0424	0.048*	
C7	0.4519 (5)	0.3035 (4)	0.9327 (3)	0.0343 (11)	
C8	0.1464 (6)	0.1917 (5)	0.9273 (3)	0.0487 (14)	
C9	0.8608 (5)	0.6408 (4)	0.9097 (3)	0.0417 (13)	
H9	0.9122	0.6038	0.9522	0.050*	
C10	0.9382 (6)	0.7197 (5)	0.8777 (3)	0.0492 (14)	
H10	1.0406	0.7343	0.8965	0.059*	
C11	0.8582 (7)	0.7770 (5)	0.8163 (4)	0.0567 (16)	
H11	0.9068	0.8322	0.7943	0.068*	
C12	0.7096 (6)	0.7528 (4)	0.7883 (3)	0.0463 (14)	
H12	0.6560	0.7903	0.7468	0.056*	
C13	0.6397 (6)	0.6717 (4)	0.8223 (3)	0.0355 (12)	
C14	0.4773 (5)	0.6390 (4)	0.7981 (3)	0.0344 (11)	
C15	0.3784 (6)	0.6848 (4)	0.7381 (3)	0.0452 (13)	
H15	0.4122	0.7385	0.7086	0.054*	
C16	0.2298 (7)	0.6503 (5)	0.7225 (3)	0.0537 (15)	
H16	0.1619	0.6808	0.6825	0.064*	
C17	0.1818 (6)	0.5702 (5)	0.7664 (4)	0.0525 (15)	
H17	0.0813	0.5461	0.7566	0.063*	
C18	0.2852 (6)	0.5264 (4)	0.8252 (3)	0.0413 (13)	
H18	0.2529	0.4721	0.8548	0.050*	
C19	0.7153 (6)	0.5993 (5)	0.5270 (4)	0.0615 (17)	
C20	0.6265 (6)	0.6901 (4)	0.5381 (4)	0.0524 (15)	
H20	0.6489	0.7359	0.5843	0.063*	
C21	0.5074 (5)	0.7060 (4)	0.4773 (3)	0.0400 (13)	
C22	0.4773 (7)	0.6359 (4)	0.4087 (4)	0.0494 (15)	
C23	0.5622 (7)	0.5498 (5)	0.4014 (4)	0.0675 (18)	
H23	0.5386	0.5034	0.3555	0.081*	
C24	0.6809 (7)	0.5306 (5)	0.4601 (4)	0.0683 (18)	
H24	0.7383	0.4712	0.4548	0.082*	
C25	0.4042 (5)	0.7933 (4)	0.4948 (3)	0.0375 (12)	
C26	0.3525 (7)	0.6563 (5)	0.3404 (4)	0.0560 (16)	
C27	0.3086 (6)	1.1676 (4)	0.6747 (3)	0.0393 (12)	
H27	0.3939	1.1437	0.7075	0.047*	
C28	0.2334 (6)	1.2493 (4)	0.7041 (3)	0.0462 (14)	
H28	0.2673	1.2800	0.7558	0.055*	
C29	0.1083 (6)	1.2848 (4)	0.6561 (3)	0.0468 (14)	
H29	0.0555	1.3397	0.6749	0.056*	
C30	0.0608 (6)	1.2382 (4)	0.5794 (3)	0.0413 (13)	
H30	−0.0245	1.2613	0.5461	0.050*	
C31	0.1409 (5)	1.1571 (4)	0.5525 (3)	0.0287 (10)	
C32	0.1032 (5)	1.1024 (4)	0.4714 (3)	0.0282 (10)	
C33	−0.0218 (5)	1.1238 (4)	0.4139 (3)	0.0365 (12)	
H33	−0.0886	1.1744	0.4250	0.044*	
C34	−0.0444 (6)	1.0686 (4)	0.3402 (3)	0.0428 (13)	
H34	−0.1275	1.0815	0.3009	0.051*	
C35	0.0546 (6)	0.9947 (4)	0.3246 (3)	0.0443 (13)	
H35	0.0409	0.9577	0.2747	0.053*	
C36	0.1748 (6)	0.9764 (4)	0.3845 (3)	0.0406 (12)	
H36	0.2423	0.9259	0.3741	0.049*	
Cl1	0.78986 (14)	0.41119 (12)	0.98373 (9)	0.0513 (4)	
Cl2	0.48929 (17)	0.94718 (12)	0.66912 (8)	0.0550 (4)	
Cu1	0.59103 (6)	0.50284 (5)	0.92407 (3)	0.03410 (19)	
Cu2	0.36310 (6)	1.00231 (5)	0.55154 (3)	0.03154 (18)	
N1	0.7149 (4)	0.6149 (3)	0.8824 (2)	0.0337 (9)	
N2	0.4303 (4)	0.5595 (3)	0.8412 (2)	0.0343 (9)	
N3	0.6704 (6)	0.0644 (4)	1.1394 (3)	0.0534 (12)	
N4	0.8394 (7)	0.5799 (6)	0.5897 (4)	0.0879 (19)	
N5	0.2640 (4)	1.1214 (3)	0.6011 (2)	0.0308 (9)	
N6	0.1989 (4)	1.0280 (3)	0.4569 (2)	0.0310 (9)	
O1	0.7845 (6)	0.1239 (4)	1.1487 (3)	0.0887 (16)	
O2	0.6673 (5)	−0.0224 (4)	1.1707 (2)	0.0695 (13)	
O3	0.0252 (5)	0.1282 (4)	0.9260 (3)	0.0770 (14)	
H3A	−0.0477	0.1540	0.8983	0.116*	
O4	0.1475 (4)	0.2682 (3)	0.8860 (2)	0.0548 (10)	
O5	0.4886 (4)	0.2789 (3)	0.8685 (2)	0.0519 (10)	
O6	0.4541 (4)	0.4004 (2)	0.96305 (19)	0.0347 (8)	
O7	0.9139 (7)	0.4957 (5)	0.5801 (4)	0.134 (3)	
O8	0.8725 (6)	0.6481 (5)	0.6464 (4)	0.109 (2)	
O9	0.4530 (4)	0.8915 (2)	0.49221 (19)	0.0348 (8)	
O10	0.2880 (4)	0.7654 (3)	0.5148 (3)	0.0580 (11)	
O11	0.3023 (5)	0.7424 (4)	0.3291 (3)	0.0736 (13)	
O12	0.3072 (7)	0.5694 (4)	0.2920 (3)	0.0926 (17)	
H12A	0.2152	0.5641	0.2813	0.139*	
O13	0.0783 (6)	0.5905 (4)	0.1749 (3)	0.0776 (14)	
O14	0.2367 (6)	0.8268 (4)	0.1645 (3)	0.0733 (13)	
H1W	0.075 (9)	0.6551 (19)	0.191 (4)	0.110*	
H2W	0.125 (8)	0.589 (5)	0.137 (3)	0.110*	
H3W	0.235 (9)	0.792 (5)	0.205 (3)	0.110*	
H4W	0.285 (8)	0.795 (5)	0.135 (3)	0.110*	

### Atomic displacement parameters (Å^2^)

**Table:**

	*U*^11^	*U*^22^	*U*^33^	*U*^12^	*U*^13^	*U*^23^
C1	0.054 (3)	0.029 (3)	0.033 (3)	0.009 (2)	0.008 (2)	0.004 (2)
C2	0.066 (4)	0.039 (3)	0.046 (3)	−0.004 (3)	0.014 (3)	0.013 (3)
C3	0.053 (4)	0.039 (3)	0.056 (4)	−0.010 (3)	0.021 (3)	0.012 (3)
C4	0.047 (3)	0.036 (3)	0.038 (3)	0.001 (2)	0.009 (2)	0.002 (2)
C5	0.047 (3)	0.024 (3)	0.035 (3)	0.003 (2)	0.014 (2)	0.000 (2)
C6	0.045 (3)	0.032 (3)	0.044 (3)	0.001 (2)	0.009 (2)	0.006 (2)
C7	0.031 (3)	0.041 (3)	0.029 (3)	0.004 (2)	0.000 (2)	0.009 (2)
C8	0.044 (3)	0.053 (4)	0.050 (3)	−0.007 (3)	0.015 (3)	−0.001 (3)
C9	0.030 (3)	0.045 (3)	0.049 (3)	0.005 (2)	0.003 (2)	0.018 (3)
C10	0.031 (3)	0.055 (4)	0.063 (4)	0.000 (3)	0.009 (3)	0.019 (3)
C11	0.055 (4)	0.056 (4)	0.065 (4)	−0.001 (3)	0.020 (3)	0.026 (3)
C12	0.042 (3)	0.051 (4)	0.050 (3)	0.011 (3)	0.013 (3)	0.027 (3)
C13	0.040 (3)	0.038 (3)	0.031 (3)	0.014 (2)	0.010 (2)	0.011 (2)
C14	0.037 (3)	0.034 (3)	0.032 (3)	0.010 (2)	0.003 (2)	0.005 (2)
C15	0.052 (4)	0.038 (3)	0.045 (3)	0.007 (3)	0.002 (3)	0.017 (2)
C16	0.048 (4)	0.051 (4)	0.055 (4)	0.008 (3)	−0.011 (3)	0.020 (3)
C17	0.039 (3)	0.049 (4)	0.063 (4)	−0.001 (3)	−0.005 (3)	0.004 (3)
C18	0.039 (3)	0.044 (3)	0.040 (3)	0.003 (3)	0.004 (2)	0.010 (2)
C19	0.042 (4)	0.057 (4)	0.088 (4)	0.013 (3)	0.010 (3)	0.042 (3)
C20	0.037 (3)	0.036 (3)	0.091 (5)	0.008 (3)	0.023 (3)	0.023 (3)
C21	0.028 (3)	0.023 (3)	0.074 (4)	0.006 (2)	0.019 (3)	0.018 (3)
C22	0.060 (4)	0.022 (3)	0.076 (4)	0.007 (3)	0.036 (3)	0.007 (3)
C23	0.076 (5)	0.057 (4)	0.078 (4)	0.021 (4)	0.028 (3)	0.013 (3)
C24	0.075 (5)	0.049 (4)	0.093 (5)	0.020 (3)	0.043 (4)	0.006 (3)
C25	0.027 (3)	0.036 (3)	0.052 (3)	0.006 (2)	0.009 (2)	0.007 (2)
C26	0.067 (4)	0.050 (4)	0.056 (4)	0.000 (3)	0.026 (3)	0.000 (3)
C27	0.034 (3)	0.045 (3)	0.037 (3)	0.003 (2)	0.001 (2)	0.010 (2)
C28	0.050 (3)	0.047 (3)	0.042 (3)	0.006 (3)	0.011 (3)	0.001 (3)
C29	0.053 (4)	0.042 (3)	0.047 (3)	0.017 (3)	0.015 (3)	−0.006 (3)
C30	0.035 (3)	0.044 (3)	0.047 (3)	0.013 (2)	0.009 (2)	0.006 (2)
C31	0.024 (2)	0.029 (3)	0.035 (3)	0.003 (2)	0.009 (2)	0.007 (2)
C32	0.029 (3)	0.025 (2)	0.035 (3)	0.005 (2)	0.012 (2)	0.013 (2)
C33	0.030 (3)	0.044 (3)	0.038 (3)	0.012 (2)	0.008 (2)	0.014 (2)
C34	0.038 (3)	0.054 (4)	0.035 (3)	0.009 (3)	0.001 (2)	0.012 (2)
C35	0.049 (3)	0.050 (3)	0.032 (3)	−0.001 (3)	0.004 (2)	−0.001 (2)
C36	0.043 (3)	0.040 (3)	0.042 (3)	0.010 (2)	0.013 (2)	0.002 (2)
Cl1	0.0335 (7)	0.0585 (9)	0.0638 (9)	0.0120 (6)	0.0058 (6)	0.0327 (7)
Cl2	0.0630 (10)	0.0602 (9)	0.0447 (8)	0.0299 (7)	0.0074 (7)	0.0236 (7)
Cu1	0.0295 (3)	0.0381 (4)	0.0360 (4)	0.0055 (3)	0.0059 (3)	0.0163 (3)
Cu2	0.0292 (3)	0.0324 (4)	0.0349 (3)	0.0129 (3)	0.0068 (3)	0.0097 (3)
N1	0.031 (2)	0.039 (2)	0.033 (2)	0.0112 (19)	0.0072 (18)	0.0143 (18)
N2	0.034 (2)	0.035 (2)	0.035 (2)	0.0071 (19)	0.0057 (18)	0.0123 (18)
N3	0.066 (4)	0.047 (3)	0.046 (3)	0.003 (3)	0.007 (3)	0.009 (2)
N4	0.072 (4)	0.083 (5)	0.101 (5)	0.010 (4)	−0.004 (4)	0.007 (4)
N5	0.032 (2)	0.030 (2)	0.032 (2)	0.0061 (18)	0.0064 (18)	0.0088 (17)
N6	0.031 (2)	0.026 (2)	0.037 (2)	0.0084 (17)	0.0067 (18)	0.0066 (18)
O1	0.066 (3)	0.090 (4)	0.101 (4)	−0.005 (3)	−0.013 (3)	0.047 (3)
O2	0.095 (4)	0.059 (3)	0.055 (3)	0.013 (3)	0.010 (2)	0.023 (2)
O3	0.048 (3)	0.087 (4)	0.093 (4)	−0.013 (3)	0.002 (2)	0.037 (3)
O4	0.047 (2)	0.056 (3)	0.063 (3)	0.004 (2)	0.010 (2)	0.021 (2)
O5	0.069 (3)	0.051 (2)	0.038 (2)	−0.001 (2)	0.0162 (19)	0.0067 (18)
O6	0.039 (2)	0.0252 (19)	0.0415 (19)	0.0053 (15)	0.0096 (15)	0.0077 (15)
O7	0.138 (5)	0.086 (4)	0.156 (6)	0.077 (4)	−0.038 (4)	0.006 (4)
O8	0.079 (4)	0.097 (4)	0.131 (5)	0.028 (3)	−0.029 (3)	−0.015 (4)
O9	0.0365 (19)	0.0251 (19)	0.045 (2)	0.0093 (15)	0.0118 (15)	0.0056 (15)
O10	0.038 (2)	0.042 (2)	0.101 (3)	0.0041 (18)	0.030 (2)	0.001 (2)
O11	0.090 (4)	0.050 (3)	0.075 (3)	0.022 (3)	0.001 (3)	−0.007 (2)
O12	0.127 (5)	0.056 (3)	0.086 (4)	0.003 (3)	0.006 (3)	−0.018 (3)
O13	0.095 (4)	0.061 (3)	0.082 (4)	0.008 (3)	0.030 (3)	0.004 (3)
O14	0.059 (3)	0.082 (3)	0.084 (4)	0.012 (2)	0.019 (3)	0.024 (3)

### Geometric parameters (Å, °)

**Table:**

C1—C2	1.366 (7)	C24—H24	0.9300
C1—C6	1.375 (7)	C25—O10	1.216 (6)
C1—N3	1.466 (7)	C25—O9	1.280 (6)
C2—C3	1.392 (8)	C26—O11	1.186 (7)
C2—H2	0.9300	C26—O12	1.321 (7)
C3—C4	1.384 (7)	C27—N5	1.330 (6)
C3—H3	0.9300	C27—C28	1.375 (7)
C4—C5	1.411 (7)	C27—H27	0.9300
C4—C8	1.487 (8)	C28—C29	1.364 (8)
C5—C6	1.388 (7)	C28—H28	0.9300
C5—C7	1.520 (6)	C29—C30	1.381 (7)
C6—H6	0.9300	C29—H29	0.9300
C7—O5	1.233 (6)	C30—C31	1.378 (6)
C7—O6	1.279 (6)	C30—H30	0.9300
C8—O4	1.206 (6)	C31—N5	1.358 (6)
C8—O3	1.322 (6)	C31—C32	1.478 (6)
C9—N1	1.336 (6)	C32—N6	1.341 (6)
C9—C10	1.374 (7)	C32—C33	1.388 (6)
C9—H9	0.9300	C33—C34	1.373 (7)
C10—C11	1.392 (8)	C33—H33	0.9300
C10—H10	0.9300	C34—C35	1.364 (7)
C11—C12	1.357 (8)	C34—H34	0.9300
C11—H11	0.9300	C35—C36	1.373 (7)
C12—C13	1.375 (7)	C35—H35	0.9300
C12—H12	0.9300	C36—N6	1.335 (6)
C13—N1	1.356 (6)	C36—H36	0.9300
C13—C14	1.487 (7)	Cl1—Cu1	2.2680 (14)
C14—N2	1.358 (6)	Cl2—Cu2	2.2509 (14)
C14—C15	1.380 (7)	Cu1—O6	1.968 (3)
C15—C16	1.371 (7)	Cu1—N1	1.992 (4)
C15—H15	0.9300	Cu1—N2	1.995 (4)
C16—C17	1.375 (8)	Cu1—O6^i^	2.331 (3)
C16—H16	0.9300	Cu2—O9	1.966 (3)
C17—C18	1.379 (7)	Cu2—N5	1.999 (4)
C17—H17	0.9300	Cu2—N6	2.014 (4)
C18—N2	1.337 (6)	Cu2—O9^ii^	2.341 (3)
C18—H18	0.9300	N3—O2	1.222 (6)
C19—C24	1.363 (7)	N3—O1	1.226 (6)
C19—N4	1.427 (8)	N4—O8	1.233 (8)
C19—C20	1.439 (8)	N4—O7	1.288 (8)
C20—C21	1.369 (8)	O3—H3A	0.8200
C20—H20	0.9300	O6—Cu1^i^	2.331 (3)
C21—C22	1.395 (8)	O9—Cu2^ii^	2.341 (3)
C21—C25	1.519 (7)	O12—H12A	0.8200
C22—C23	1.358 (8)	O13—H1W	0.834 (10)
C22—C26	1.493 (9)	O13—H2W	0.838 (10)
C23—C24	1.356 (7)	O14—H3W	0.834 (10)
C23—H23	0.9300	O14—H4W	0.831 (10)
			
C2—C1—C6	123.3 (5)	N5—C27—C28	122.5 (5)
C2—C1—N3	118.9 (5)	N5—C27—H27	118.7
C6—C1—N3	117.8 (5)	C28—C27—H27	118.7
C1—C2—C3	117.1 (5)	C29—C28—C27	119.0 (5)
C1—C2—H2	121.5	C29—C28—H28	120.5
C3—C2—H2	121.5	C27—C28—H28	120.5
C4—C3—C2	122.4 (5)	C28—C29—C30	119.3 (5)
C4—C3—H3	118.8	C28—C29—H29	120.3
C2—C3—H3	118.8	C30—C29—H29	120.3
C3—C4—C5	118.4 (5)	C31—C30—C29	119.5 (5)
C3—C4—C8	121.8 (5)	C31—C30—H30	120.2
C5—C4—C8	119.8 (5)	C29—C30—H30	120.2
C6—C5—C4	119.7 (5)	N5—C31—C30	120.7 (4)
C6—C5—C7	114.7 (4)	N5—C31—C32	114.7 (4)
C4—C5—C7	125.6 (4)	C30—C31—C32	124.6 (4)
C1—C6—C5	119.2 (5)	N6—C32—C33	121.6 (4)
C1—C6—H6	120.4	N6—C32—C31	114.8 (4)
C5—C6—H6	120.4	C33—C32—C31	123.7 (4)
O5—C7—O6	124.9 (5)	C34—C33—C32	118.4 (5)
O5—C7—C5	118.6 (5)	C34—C33—H33	120.8
O6—C7—C5	116.0 (4)	C32—C33—H33	120.8
O4—C8—O3	123.2 (6)	C35—C34—C33	120.2 (5)
O4—C8—C4	123.3 (5)	C35—C34—H34	119.9
O3—C8—C4	113.5 (5)	C33—C34—H34	119.9
N1—C9—C10	122.9 (5)	C34—C35—C36	118.4 (5)
N1—C9—H9	118.6	C34—C35—H35	120.8
C10—C9—H9	118.6	C36—C35—H35	120.8
C9—C10—C11	117.7 (5)	N6—C36—C35	122.8 (5)
C9—C10—H10	121.1	N6—C36—H36	118.6
C11—C10—H10	121.1	C35—C36—H36	118.6
C12—C11—C10	120.3 (5)	O6—Cu1—N1	175.05 (14)
C12—C11—H11	119.9	O6—Cu1—N2	94.41 (15)
C10—C11—H11	119.9	N1—Cu1—N2	81.37 (16)
C11—C12—C13	118.9 (5)	O6—Cu1—Cl1	90.06 (10)
C11—C12—H12	120.6	N1—Cu1—Cl1	94.74 (11)
C13—C12—H12	120.6	N2—Cu1—Cl1	162.02 (12)
N1—C13—C12	122.0 (5)	O6—Cu1—O6^i^	78.33 (13)
N1—C13—C14	114.0 (4)	N1—Cu1—O6^i^	99.69 (14)
C12—C13—C14	124.0 (4)	N2—Cu1—O6^i^	98.93 (14)
N2—C14—C15	121.1 (5)	Cl1—Cu1—O6^i^	99.03 (9)
N2—C14—C13	114.4 (4)	O9—Cu2—N5	174.18 (14)
C15—C14—C13	124.5 (5)	O9—Cu2—N6	93.77 (14)
C16—C15—C14	119.4 (5)	N5—Cu2—N6	81.05 (15)
C16—C15—H15	120.3	O9—Cu2—Cl2	90.70 (10)
C14—C15—H15	120.3	N5—Cu2—Cl2	95.02 (11)
C15—C16—C17	119.6 (5)	N6—Cu2—Cl2	162.80 (12)
C15—C16—H16	120.2	O9—Cu2—O9^ii^	79.29 (13)
C17—C16—H16	120.2	N5—Cu2—O9^ii^	98.40 (13)
C16—C17—C18	118.8 (5)	N6—Cu2—O9^ii^	94.85 (13)
C16—C17—H17	120.6	Cl2—Cu2—O9^ii^	102.30 (9)
C18—C17—H17	120.6	C9—N1—C13	118.2 (4)
N2—C18—C17	122.2 (5)	C9—N1—Cu1	126.5 (3)
N2—C18—H18	118.9	C13—N1—Cu1	115.3 (3)
C17—C18—H18	118.9	C18—N2—C14	118.9 (4)
C24—C19—N4	120.0 (6)	C18—N2—Cu1	126.2 (3)
C24—C19—C20	121.8 (6)	C14—N2—Cu1	114.9 (3)
N4—C19—C20	118.2 (6)	O2—N3—O1	122.5 (5)
C21—C20—C19	116.8 (6)	O2—N3—C1	119.2 (5)
C21—C20—H20	121.6	O1—N3—C1	118.3 (5)
C19—C20—H20	121.6	O8—N4—O7	124.9 (7)
C20—C21—C22	120.1 (5)	O8—N4—C19	117.6 (7)
C20—C21—C25	115.7 (5)	O7—N4—C19	117.3 (7)
C22—C21—C25	123.8 (5)	C27—N5—C31	119.0 (4)
C23—C22—C21	121.2 (6)	C27—N5—Cu2	126.4 (3)
C23—C22—C26	118.5 (6)	C31—N5—Cu2	114.6 (3)
C21—C22—C26	120.2 (5)	C36—N6—C32	118.6 (4)
C22—C23—C24	120.8 (7)	C36—N6—Cu2	126.6 (3)
C22—C23—H23	119.6	C32—N6—Cu2	114.7 (3)
C24—C23—H23	119.6	C8—O3—H3A	109.5
C23—C24—C19	119.3 (6)	C7—O6—Cu1	113.7 (3)
C23—C24—H24	120.4	C7—O6—Cu1^i^	141.6 (3)
C19—C24—H24	120.4	Cu1—O6—Cu1^i^	101.67 (13)
O10—C25—O9	125.3 (5)	C25—O9—Cu2	116.4 (3)
O10—C25—C21	118.3 (4)	C25—O9—Cu2^ii^	142.6 (3)
O9—C25—C21	116.2 (4)	Cu2—O9—Cu2^ii^	100.71 (13)
O11—C26—O12	123.2 (6)	C26—O12—H12A	109.5
O11—C26—C22	123.6 (6)	H1W—O13—H2W	108 (3)
O12—C26—C22	113.1 (6)	H3W—O14—H4W	110 (3)

Symmetry codes: (i) −*x*+1, −*y*+1, −*z*+2; (ii) −*x*+1, −*y*+2, −*z*+1.

### Hydrogen-bond geometry (Å, °)

**Table:**

*D*—H···*A*	*D*—H	H···*A*	*D*···*A*	*D*—H···*A*
O3—H3A···O14^iii^	0.82	1.87	2.664 (6)	164
O12—H12A···O13	0.82	2.03	2.611 (8)	127
O13—H1W···O14	0.83 (1)	2.64 (5)	3.229 (7)	129 (5)
O13—H2W···Cl1^iv^	0.84 (1)	2.34 (2)	3.168 (5)	169 (8)
O14—H3W···O11	0.83 (1)	2.20 (3)	2.992 (7)	158 (8)
O14—H4W···O5^iv^	0.83 (1)	2.30 (5)	3.010 (6)	143 (7)

Symmetry codes: (iii) −*x*, −*y*+1, −*z*+1; (iv) −*x*+1, −*y*+1, −*z*+1.
